# Whole genome sequencing data of multiple individuals of Pakistani descent

**DOI:** 10.1038/s41597-020-00664-2

**Published:** 2020-10-13

**Authors:** Shahid Y. Khan, Muhammad Ali, Mei-Chong W. Lee, Zhiwei Ma, Pooja Biswas, Asma A. Khan, Muhammad Asif Naeem, Saima Riazuddin, Sheikh Riazuddin, Radha Ayyagari, J. Fielding Hejtmancik, S. Amer Riazuddin

**Affiliations:** 1 The Wilmer Eye Institute, Johns Hopkins University School of Medicine, Baltimore, MD 21287 USA; 2 Department of Computer Science, San José State University, San José, CA 95192 USA; 3 Ophthalmic Genetics and Visual Function Branch, National Eye Institute, National Institutes of Health, Bethesda, MD 20892 USA; 4 Shiley Eye Institute, University of California San Diego, La Jolla, CA 92093 USA; 5 National Centre of Excellence in Molecular Biology, University of the Punjab, Lahore, 53700 Pakistan; 6 Department of Otorhinolaryngology-Head & Neck Surgery, University of Maryland School Medicine, Baltimore, MD 21201 USA; 7 Allama Iqbal Medical College, University of Health Sciences, Lahore, 54550 Pakistan; 8 Department of Molecular Biology, Shaheed Zulfiqar Ali Bhutto Medical University, Islamabad, 44080 Pakistan

**Keywords:** Next-generation sequencing, Comparative genomics

## Abstract

Here we report whole genome sequencing of four individuals (H3, H4, H5, and H6) from a family of Pakistani descent. Whole genome sequencing yielded 1084.92, 894.73, 1068.62, and 1005.77 million mapped reads corresponding to 162.73, 134.21, 160.29, and 150.86 Gb sequence data and 52.49x, 43.29x, 51.70x, and 48.66x average coverage for H3, H4, H5, and H6, respectively. We identified 3,529,659, 3,478,495, 3,407,895, and 3,426,862 variants in the genomes of H3, H4, H5, and H6, respectively, including 1,668,024 variants common in the four genomes. Further, we identified 42,422, 39,824, 28,599, and 35,206 novel variants in the genomes of H3, H4, H5, and H6, respectively. A major fraction of the variants identified in the four genomes reside within the intergenic regions of the genome. Single nucleotide polymorphism (SNP) genotype based comparative analysis with ethnic populations of 1000 Genomes database linked the ancestry of all four genomes with the South Asian populations, which was further supported by mitochondria based haplogroup analysis. In conclusion, we report whole genome sequencing of four individuals of Pakistani descent.

## Abstract (summary)

**Table:** 

Measurement(s)	SNV • genome
Technology Type(s)	whole genome sequencing • DNA sequencing
Factor Type(s)	individual
Sample Characteristic - Organism	Homo sapiens
Sample Characteristic - Location	Pakistan

Machine-accessible metadata file describing the reported data: 10.6084/m9.figshare.12642761

## Background & Summary

The completion of Human Genome Project ignited several large scale efforts to characterize variations in the human genome, which led to a comprehensive catalog of the common variants including single-nucleotide polymorphisms (SNPs) and insertions/deletions (indels), across the entire human genome^1,2^. Population-based genome reference datasets played an important role in elucidation of rare variants in specific populations^3,4^. So far, comprehensive genome reference datasets have been reported for African, Japanese, Korean, and Chinese populations^5–8^.

Advancements in next-generation sequencing technologies have impelled the development of a comprehensive catalog of genetic variants from different ethnic populations^9–15^. The 1000 Genomes Project reports human genetic variation profiles from 26 ethnic populations, including one Pakistani (Punjabi), two Indian (Gujarati and Telugu), one Bangladeshi (Bengali), and one Sri Lankan (Tamil) population—all descendants of the Indian subcontinent^15^.

Additionally, independent groups have recently published two Indian and two Pakistani genomes with an overall 25–30× sequencing coverage^16–19^. Recently, the GenomeAsia 100 K project reported genomes of 1,739 individuals, including 113 individuals of Pakistani origin (https://browser.genomeasia100k.org). We previously reported the whole genome sequencing of two Pakistani individuals^20^. Here, we report whole genome sequencing of four individuals of Pakistani descent.

## Methods

### Sample collection

The protocol for this study was approved by the Institutional Review Board of the Johns Hopkins University School of Medicine (Baltimore, MD), the National Centre of Excellence in Molecular Biology (Lahore, Pakistan), and the National Eye Institute (Bethesda, MD). The participating members provided informed written consent consistent with the tenets of the Declaration of Helsinki. A small aliquot (~10 ml) of a blood sample was collected from each individual and genomic DNA was extracted as previously described^20^.

### Library preparation and next-generation sequencing

Whole genome sequencing was performed using the Illumina HiSeq X10 (Illumina, San Diego, CA, USA). Briefly, 1.0–2.0 µg of fragmented gDNA was used to prepare paired-end libraries with the TruSeq DNA PCR-Free Library Preparation Kit for four samples (H3, H4, H5, and H6) according to the manufacturer’s instructions (Illumina Inc., San Diego, CA). All four libraries were sequenced using Illumina HiSeq X10 in paired-end fashion (2 × 150 bp; Illumina Inc.). The base calls were assigned through Illumina Real-Time Analysis software (Ver. 1.17.20) and binary base call (BCL) files were converted to flat-file format (qseq.txt) using Illumina BCL Converter software (Ver. 1.9.4).

### Bioinformatics analysis

Paired-end raw reads were aligned to the human reference genome (GRCh38.p13) using Burrows-Wheeler Aligner-MEM (BWA-MEM; Ver. 0.7.17-r1188) without ALT-aware mode^21^. The quality of the read alignments was examined using CollectAlignmentSummaryMetrics from Picard Tools (Ver. 2.19.0; http://broadinstitute.github.io/picard). The duplicate reads were removed from the mapped reads using MarkDuplicates from Picard Tools. The variants including SNPs and indels were called using the Genome Analysis Tool Kit (GATK; Ver. 4.0) best-practices^12,22^. Briefly, the recalibration of base qualities of input reads was performed using GATK tools (BaseRecalibrator and ApplyBQSR). Subsequently, the SNPs, indels, and genotype of variants were identified using multiple tools i.e. HaplotypeCaller (in GVCF mode), GenotypeGVCFs, and VCFtools (Ver. 0.1.15)^23^. Alignment metrics were generated using CollectAlignmentSummaryMetrics and CollectInsertSizeMetrics from Picard Tools. Genome-wide read coverage was generated using Bedtools (Ver. 2.26.0)^24^.

### Variant filtering and annotation

The variants identified through the GATK tool kit were further screened using the high-confidence regions characterized by Genome in a Bottle (GIAB) database^25^. The variants aligned within the large segmental duplication regions of the human genome were discarded while variants mapped to the high-confidence regions of GIAB were used in downstream analyses including Venn diagram generation using VennPainter^26^. **Note:** An allele (variant) with a minimum of 40% of the total reads mapped to reference allele is considered authentic. The filtered variants were annotated using clinEff (Ver. 1.0 h; http://www.dnaminer.com/clineff.html), a professional version of SNPEff^27^, designed for the prediction of functional effects of variants.

### Variant calling

The CNVnator (Ver. 0.4.1) algorithm was used for the identification of copy number variations (CNVs) with a bin size of 1,000 and 10,0000^28^. The GIAB filtered variants (SNPs) were imported into the CNV analysis pipeline for plotting the B-allele frequency (BAF) along the read depths for all deletion and duplication events.

### Ancestry prediction

The ancestral roots of H3, H4, H5, and H6 were examined using the algorithms of Peddy (Ver. 0.3.5)^29^. The study utilized the high-performance computational capabilities of the Biowulf Linux cluster at the National Institutes of Health, Bethesda, MD. PCA plots were created using SNPs genotype information obtained from VCF (variant call format) files (from whole genome sequencing data of H3, H4, H5, and H6) and comparing it with combined ethnic populations from the 1000 Genomes dataset.

In parallel, ancestral roots of H3, H4, H5, and H6 were examined through a comparative analysis with genomes of five different ethnic populations within the 1000 Genomes database. We randomly selected 96 samples from African, Ad Mixed American, East Asian, European, and South Asian populations for comparative analysis by the bcftools-isec algorithm. These variants from 1000 Genomes database and four genomes in VCF format were converted to BCF using bcftools (Ver. 1.8). The BCF files were converted to PLINK format using PLINK (Ver. 1.90b6.18) and PLINK algorithms were used to filter the variants to generate a list of markers in approximate linkage equilibrium for PCA analysis.

### Haplogroup analysis

The mitochondrial sequencing reads were mapped to the revised Cambridge reference sequence (rCRS) of the human mitochondrial genome^30^. Mitochondrial variants were identified using GATK (Ver. 4.0) best practices and are classified into phylogenetic clusters in the haplogroup analysis using HaploGrep 2 (Ver. 2.1.25)^31^, with Kulczynski measure and Phylotree (build 17). The Y chromosomal haplogroup analysis for all four genomes was performed using Yleaf^32^.

## Data Records

The next-generation whole genome sequencing raw reads of H3, H4, H5, and H6 have been deposited in the NCBI Sequence Read Archive (SRA) with the accession number PRJNA596295^33^. The chromosomal distribution of the variants identified in H3, H4, H5, and H6 genomes is available at figshare^34^.

## Technical Validation

The next-generaton whole genome sequencing generated 1344.74, 1110.55, 1200.77, and 1142.35 million total reads for H3, H4, H5, and H6, respectively (Table 1)^33^. Quality control (QC) examination of the sequencing reads revealed that >99% of the sequencing data yielded a PHRED score of 30 or above (PHRED score of 30 represents the probability of 0.001 that the base call is wrong). Subsequent to QC examination and the removal of PCR duplicates (~10–18% of reads were marked duplicates and subsequently removed in downstream analysis), the majority of the reads (>99% of reads with a PHRED score ≥ 30) mapped to reference human genome (GRCh38.p13; Table 1). Mapping of the paired-end reads identified an estimated mean insert size of 390 bp in all four genomes. Majority of the total mapped reads showed paired-end alignment, resulting in 1084.92, 894.73, 1068.62, and 1005.77 million mapped reads corresponding to 162.73, 134.21, 160.29, and 150.86 Gb sequence data and 52.49x, 43.29x, 51.70x, and 48.66x average coverage for the genomes of H3, H4, H5, and H6, respectively (Table 1).

**Table 1:** Summary of the next-generation whole genome sequencing data.

Sample ID	Total reads (10^6^)	Total reads w/o PCR duplication (10^6^)	Mapped reads (10^6^)	Mapped reads (%)	Sequenced bases (Gb)	Mean depth (x)
H3	1344.74	1091.08	1084.92	99.44	162.73	52.49
H4	1110.55	899.761	894.73	99.44	134.21	43.29
H5	1200.77	1075.53	1068.62	99.36	160.29	51.70
H6	1142.35	1012.29	1005.77	99.36	150.86	48.66

**Fig. 1:**
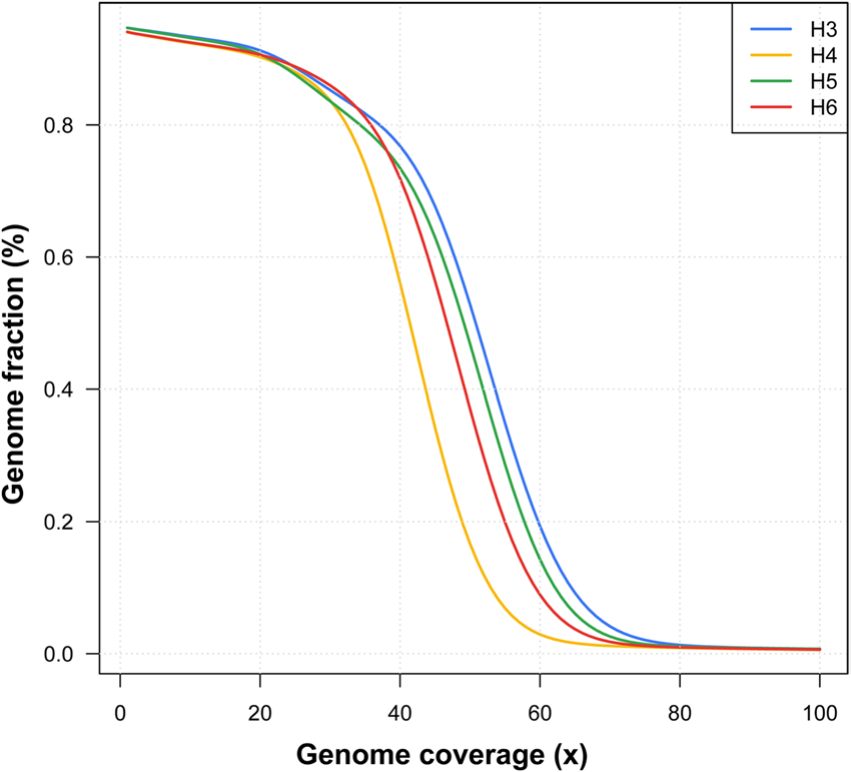
Histogram depicting the sequence coverage of the genomes of H3, H4, H5, and H6. The x- and y-axes represent the genome coverage (sequencing depth) and genome fraction (%), respectively. The blue, yellow, green, and red colors represent the genomes of H3, H4, H5, and H6, respectively.

The evaluation of sequencing reads revealed that a significant fraction of the genomes of H3, H4, H5, and H6 exhibited 30–60x read coverage (Fig. 1 and Table 2). Importantly, 5–6% of the genomes of H3, H4, H5, and H6 were not captured, representing 0x read coverage while approximately, 1%, 2%, and 5% of four genomes exhibited 1–10x, 10–20x, and 20–30x read coverage, respectively (Fig. 1 and Table 2). A minor fraction i.e. <1% of the genomes of H3, H4, H5, and H6 exhibited 80–100x read coverage (Fig. 1 and Table 2).

**Table 2:** The genome coverage of whole genome sequencing data.

Genome Coverage	Fraction of H3 genome (%)	Fraction of H4 genome (%)	Fraction of H5 genome (%)	Fraction of H6 genome (%)
0x	5.44	6.17	5.48	6.15
1–10x	1.36	1.61	1.50	1.54
10–20x	2.31	2.30	2.80	1.98
20–30x	6.29	7.58	7.44	5.10
30–40x	9.18	30.6	10.95	15.97
40–50x	26.09	37.66	28.65	36.02
50–60x	32.50	11.56	31.04	25.76
60–70x	13.27	1.38	9.88	5.88
70–80x	2.31	0.27	1.21	0.67
80–90x	0.37	0.17	0.22	0.19
90–100x	0.16	0.12	0.14	0.13
>100x	0.72	0.58	0.69	0.61

Sequence analysis of the genome of H3 revealed a total of 3,529,659 variants including 3,035,369 SNPs and 494,290 indels. The SNPs were annotated against dbSNP (Ver. 150) that identified 7,553 novel variants (0.21% of the total variants) in the genome of H3^34^. A total of 494,290 indels including 34,869 novel indels (7.05% of the total indels) were identified in the H3 genome^34^.

Sequence analysis of the genome of H4 identifed 3,478,495 total variants including 2,996,403 SNPs and 482,092 indels while annotation of the SNPs identified 6,631 novel SNPs (0.19% of the total variants) in the genome of H4^34^. A total of 482,092 indels including 33,193 novel indels (6.88% of the total indels) were identified in the genome of H4^34^. Sequence analysis of the genome of H5 identifed 3,407,895 total variants including 2,983,279 SNPs and 424,616 indels while annotation of the SNPs identified 5,560 novel SNPs (0.16% of the total variants) in the genome of H5^34^. A total of 424,616 indels including 23,039 novel indels (5.42% of the total indels) were identified in the genome of H5^34^. Finally, sequence analysis of the genome of H6 identifed 3,426,862 total variants including 2,972,863 SNPs and 453,999 indels while annotation of the SNPs identified 6,703 novel SNPs (0.19% of the total variants) in the genome H6^34^. A total of 453,999 indels including 28,503 novel indels (6.28% of the total indels) were identified in the genome of H6^34^.

**Fig. 2:**
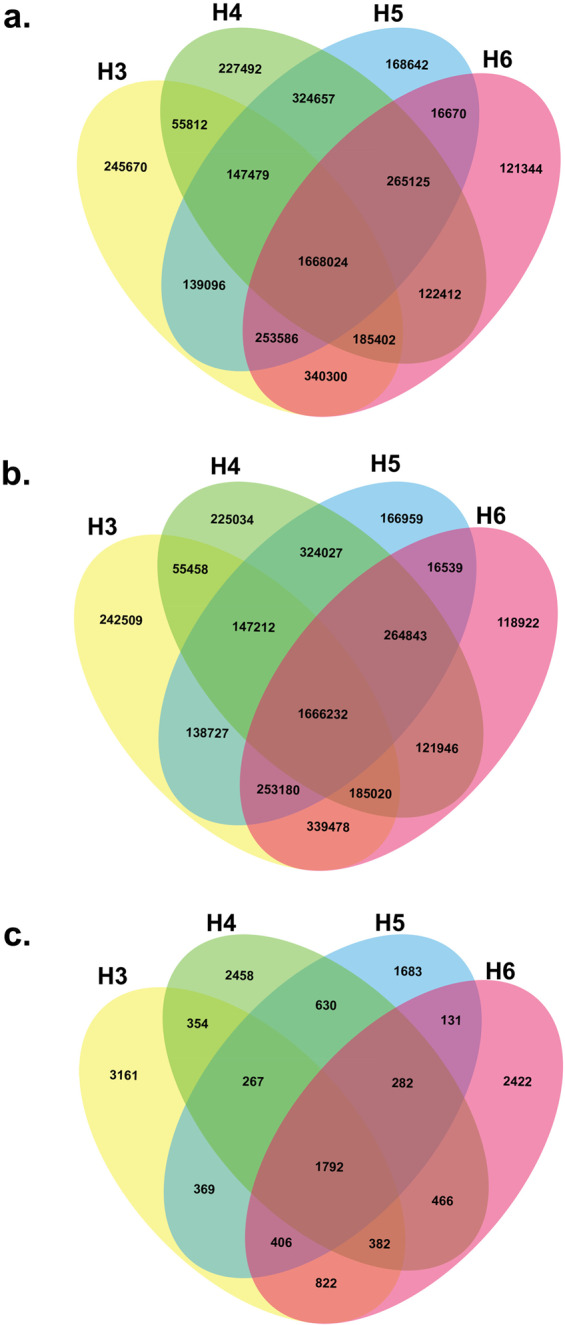
Venn diagram illustration of the overlapping variants characterized by the Genome in a Bottle (GIAB) database in the genomes H3, H4, H5, and H6. (**a**) Venn diagram illustrating all variants, (**b**) Venn diagram illustrating all known variants, and (**c**) Venn diagram illustrating all novel variants identified in the high-confidence regions characterized by the GIAB database in H3, H4, H5, and H6 genomes. **Note:** Yellow, green, blue, and pink represent variants in the genomes of H3, H4, H5, and H6, respectively, whereas darker shades represent common variants in these genomes.

Importantly, we identified a total of 1,668,024 variants including 1,666,232 variants reported previously and 1,792 novel SNPs common in the four genomes (Fig. 2a–c). Altogether, the variants common in the four genomes constitute nearly half of the total variants identified in each genome.

We examined the putative effect of the variants based on their location in the genome (exon, intron, etc.), functional impact (high, moderate, and low), and classification (synonymous vs. non-synonymous), etc. The analysis revealed that intergenic regions harbor the majority of SNPs consistent with the GIAB high-confidence variants. Furthermore, in contrast to intergenic variants, fewer variants were identified in the exons, splice site, and untranslated regions (UTRs) of the genome. Furthermore, >3 K, >82 K, and >28 K variants present in all four genomes were predicted to exhibit a putative high, moderate, and low impact, respectively.

We used CNVnator, an algorithm to characterize copy number variations (CNVs), to examine structural variants in the genomes of H3, H4, H5, and H6. The analysis identified a total of 4,269 copy number variation regions (CNVRs) common in four genomes, covering 305.95 Mb (9.53%) of the reference human genome (GRCh38.p13).

**Fig. 3:**
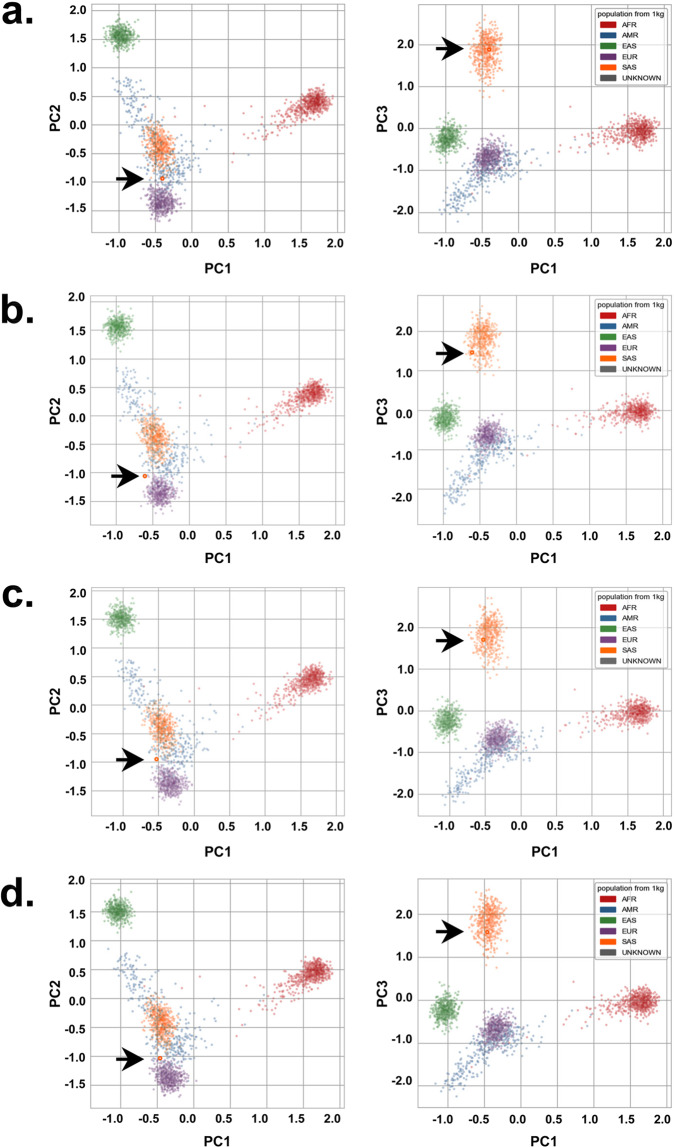
Examining the ancestral roots of H3, H4, H5, and H6 through SNP-genotype comparative analysis with the combined ethnic populations of the 1000 Genomes dataset. The arrows point to (**a**) H3, (**b**) H4, (**c**) H5 and (**d**) H6 shown as red circles in the principal component analysis (PCA) plots with South Asian populations in principal components 1 and 3 (PC1 and PC3) and between South Asian, and European populations in principal component 2 (PC2). The x-axis represents PC1 while the y- and the z-axis represent PC2 and PC3, respectively. **Note:** South Asian: SAS; African: AFR; Ad Mixed American: AMR; and East Asian: EAS.

Although H3, H4, H5, and H6 belong to the Punjabi ethnic group of Pakistani population suggesting a close ancestral relationship with South Asian populations, we sought of genomic evidence to confirm the ancestral roots of the four genomes. We compared the SNP genotypes of H3, H4, H5, and H6 to the combined population of the 1000 Genomes project by the Peddy algorithm. The analysis localized the all four genomes within South Asian populations in principal component 1 and 3 (PC1 and PC3) (Fig. 3a–d; arrows pointing to samples shown as red circles in PCA plots) and on the edge of the South Asian populations in principal component 2 (PC2) towards the European populations (Fig. 3a–d). The localization of H3, H4, H5, and H6 in PC2 suggests some ancestral link with European populations.

**Fig. 4:**
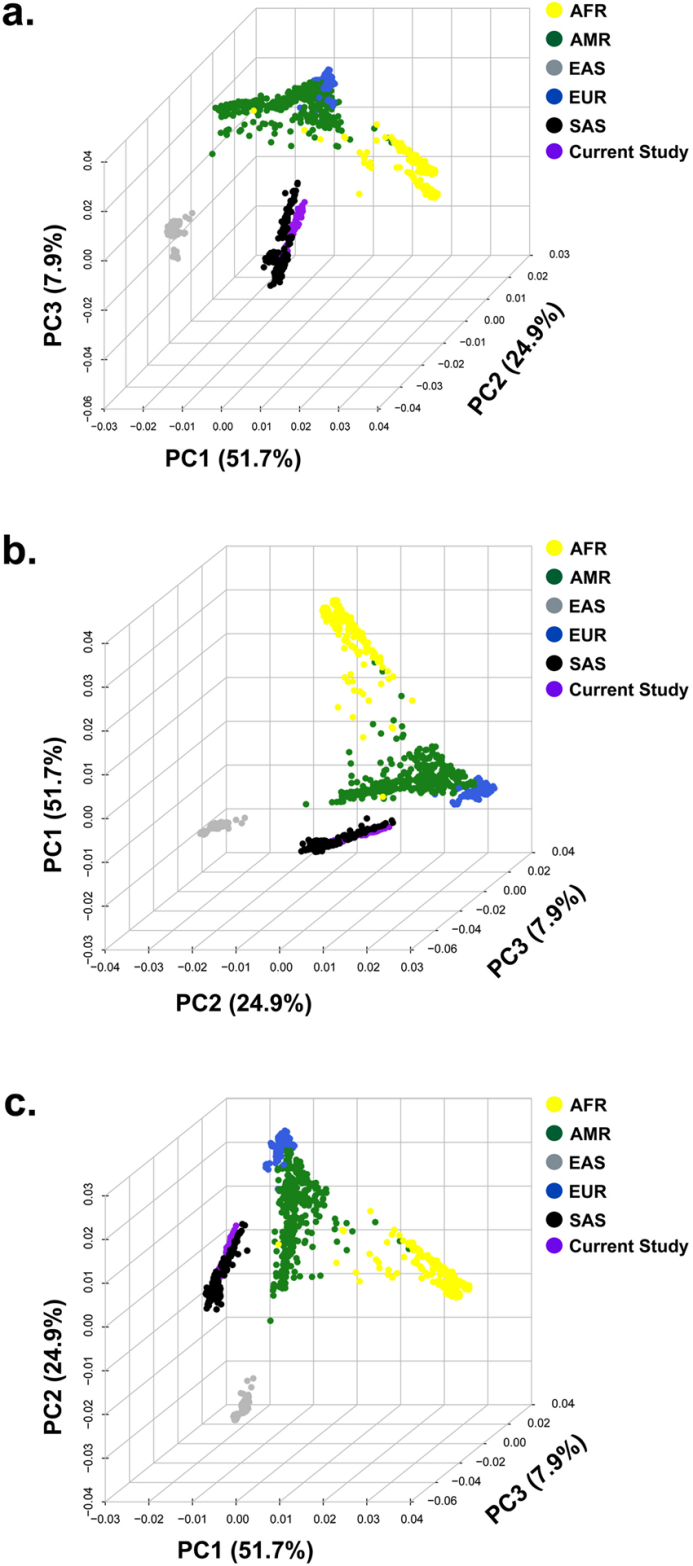
Investigating the ancestral origins of H3, H4, H5, and H6 by SNP-genotype comparative analysis with 96 random genomes from five different ethnic groups within the 1000 Genomes dataset. (**a**–**c**) Principal component analysis (PCA) plots illustrate three different angles of principal component # 1 (PC1), principal component # 2 (PC2), and principal component # 3 (PC3), respectively. The x-, y-, and z-axes depict the three largest components of the total variation in the percent of PC1, PC2, and PC3, respectively. The analysis illustrates an overlapping of four genomes with the South Asian population. **Note:** South Asian: SAS; African: AFR; Ad Mixed American: AMR; East Asian: EAS; and European: EUR.

In parallel, we performed an additional comparative analysis of the four genomes with the genomes of five different ethnic populations in the 1000 Genomes database. The analysis identified >94% overlap of variants in the genomes of H3, H4, H5, and H6 with South Asian populations (Table 3) with a small number of variants in the genomes of  H3 (157,110), H4 (166,633), H5 (159,515), and H6 (163,635) genomes not present in South Asian populations (Table 3). We identified > 92% overlap of variants in the genomes of H3, H4, H5, and H6 with both European and Ad Mixed American populations (Table 3). Likewise, we identified > 88% and > 90% overlap of variants in the genomes of H3, H4, H5, and H6 with East Asian and African populations, respectively (Table 3). These data support the notion that H3, H4, H5, and H6 have a close ancestral relationship with South Asian populations (Fig. 4a–c).

**Table 3:** The variants present in H3, H4, H5, and H6 genomes overlapping with 1000 Genomes ethnic populations.

1000 Genomes populations	African			
Sample ID	common	unique	% common	% unique
H3	2758075	277294	90.865	9.135
H4	2716661	245307	90.664	9.336
H5	2744407	238872	91.993	8.007
H6	2699787	273076	90.814	9.186
**1000 Genomes populations**	**South Asian**			
**Sample ID**	**common**	**unique**	**% common**	**%unique**
H3	2878259	157110	94.824	5.176
H4	2829770	166633	94.439	5.561
H5	2823764	159515	94.653	5.347
H6	2809228	163635	94.496	5.504
**1000 Genomes populations**	**East Asian**			
**Sample ID**	**common**	**unique**	**% common**	**% unique**
H3	2700367	335002	88.963	11.366
H4	2654822	341581	88.600	11.400
H5	2687681	295598	90.092	9.908
H6	2644954	327909	88.970	11.030
**1000 Genomes populations**	**European**			
**Sample ID**	**common**	**unique**	**% common**	**% unique**
H3	2794183	213551	92.054	7.946
H4	2753320	208648	91.888	8.112
H5	2778513	206924	93.136	6.864
H6	2733120	208367	91.936	8.064
**1000 Genomes populations**	**Admixed American**			
**Sample ID**	**common**	**unique**	**% common**	**% unique**
H3	2789486	245883	91.899	8.101
H4	2748836	247567	91.738	8.262
H5	2774780	208499	93.011	6.989
H6	2729535	243328	91.815	8.185

To further confirm the results of SNP genotype based analysis, we performed mitochondria and Y chromosome based haplogroup analysis. The mitochondria genome analysis revealed M35b haplogroup in the H3 genome and M6 haplogroup in H4, H5, and H6 genomes. Both mitochondrial haplogroups (M35b and M6) have been mainly identified in South Asian populations^35,36^. The Y chromosome analysis identified G1a1b2a haplogroup in H3 and H5 genomes, suggesting a Middle Eastern origin. Taken together, the mitochondria haplogroup based analyses support the results of the SNP genotype based analysis and strengthen the notion that H3, H4, H5, and H6 have a close ancestral relationship with South Asian populations.

In conclusion, we have completed next-generation based whole genome sequencing of four individuals from a family of Pakistani descent. Importantly, nearly 1% of the total variants identified in each of the four genomes are novel and have not been reported previously. To the best of our knowledge, this is the first report of whole genome sequencing of four individuals from a family.
